# Determination of the Normative Value of Fetal Left Brachiocephalic Vein Diameter Using Ultrasound in a Tertiary Care Hospital in Eastern India

**DOI:** 10.7759/cureus.70258

**Published:** 2024-09-26

**Authors:** Debabrata Maitra, Suman Hela, Saumyen De, Sandip K Mandal, Tanmoy Bhowmick, Sanghamitra Mandal, Mrinmoy Sutradhar, Ayan Sarkar

**Affiliations:** 1 Radiodiagnosis, College of Medicine and Sagore Dutta Hospital, Kolkata, IND; 2 Paediatrics, College of Medicine and Sagore Dutta Hospital, Kolkata, IND

**Keywords:** basic fetal echocardiography, fetal biometric parameters, fetal left brachiocephalic vein, prenatal diagnosis, second-trimester fetal anomaly scan

## Abstract

Introduction: Fetal ultrasound plays a vital role in prenatal care by offering key insights into fetal growth and facilitating the early identification of congenital abnormalities. Among various anatomical structures, the fetal left brachiocephalic vein (FLBCV) plays a significant role in fetal circulation, yet normative data for FLBCV dimensions are limited, particularly in diverse populations. This study aims to establish normative values for FLBCV diameter in fetuses between 18 and 23 weeks of gestation in a tertiary care hospital in Eastern India. Such data are essential for accurate clinical assessment and early diagnosis of potential congenital anomalies.

Methodology: This descriptive study was conducted at the College of Medicine and Sagore Dutta Hospital, Kolkata, India, from January to December 2022. The study included 287 singleton pregnancies between 18 and 23 weeks of gestation, excluding cases with maternal or fetal conditions that could affect the measurements. LBCV diameter was measured using transabdominal ultrasound, and the normative values were established using percentile charts. Correlation analyses were performed to assess the relationship between FLBCV diameter and various fetal and maternal parameters. Linear regression analysis was done to identify significant predictors of FLBCV diameter.

Results: FLBCV diameter showed a consistent increase with gestational age, ranging from a median of 1.87 mm at 18 completed weeks to 2.27 mm at 22 completed weeks. The 95th percentile values exhibited more pronounced growth, particularly between 18 and 20 weeks. Significant positive correlations were observed between FLBCV diameter and fetal biometric parameters such as biparietal diameter, head circumference, abdominal circumference, and femur length. Head circumference emerged as the most significant predictor (p=0.007) of FLBCV diameter in the linear regression model.

Conclusion: This study provides essential normative data on FLBCV diameter for the Indian population, offering a valuable reference for clinicians in prenatal diagnostics. The incorporation of FLBCV measurement in basic fetal echocardiography, which is a part of the second-trimester fetal anomaly scan, could enhance the detection of congenital anomalies, contributing to improved prenatal care.

## Introduction

Fetal ultrasound has long been an indispensable tool in prenatal care, providing critical insights into fetal development and aiding in the early detection of congenital anomalies. Among the various anatomical structures that can be evaluated by ultrasound, the fetal left brachiocephalic vein (FLBCV) plays an important role in fetal circulation, formed by the union of the left internal jugular vein with the left subclavian vein [1], draining into the superior vena cava. The FLBCV passes almost horizontally through the superior mediastinum, anterior to the aortic arch, and posterior to the thymus [2]. The LBCV can be seen and imaged on prenatal ultrasound in the upper mediastinal oblique transverse plane, cranial to the three-vessel trachea (3VT) view [3,4].

The best time to perform a thorough transabdominal fetal echocardiography is between 18 and 22 weeks of gestation [5]. The basic fetal echocardiography conducted during the second-trimester fetal anomaly scan includes views depicting situs, cardiac four chambers, ventricular outflow tracts, three-vessel view, and 3VT view. Assessment of the FLBCV is not included in basic fetal echocardiographic views to date [5]. However, dilation, absence, or aberrant course of the FLBCV points toward various congenital cardiac and extracardiac anomalies [1,6], such as total anomalous pulmonary venous connection (TAPVC), coarctation of the aorta, cerebral arteriovenous malformation, etc.

Despite its significance, normative data for FLBCV diameter remain limited, particularly among diverse populations. Establishing standard reference values is essential for accurate clinical assessment and diagnosis. This study aims to determine the normative value of FLBCV diameter using ultrasound among fetuses in a tertiary care hospital in Eastern India, which, in turn, could lead to better-informed interventions and more tailored healthcare strategies, ultimately improving maternal and fetal outcomes.

## Materials and methods

Study design, setting, and population

This descriptive study was conducted at the College of Medicine and Sagore Dutta Hospital (CMSDH), Kolkata, from January 1 to December 31, 2022, in the Department of Radiology, after obtaining approval from the Institutional Ethics Committee of the College of Medicine and Sagore Dutta Hospital, as indicated by the clearance certificate bearing memo number CMSDH/IEC/133/12-2018 dated December 22, 2018. The study population consisted of the fetuses of expecting mothers attending the obstetrics outpatient department and referred for ultrasound. Inclusion criteria were expecting mothers with singleton pregnancies, between 18 and 23 weeks of gestation, willing to participate in this study. Exclusion criteria were maternal age less than 18 years or more than 35 years, associated maternal medical conditions (e.g., maternal BMI ≥35 kg/m2, diabetes, hypertension, epilepsy, chronic renal or cardiac disease, autoimmune disease), multiple pregnancies, polyhydramnios or oligohydramnios, fetal congenital abnormalities, and refusal to participate in this study. The FLBCV was assessed in 300 consecutive singleton pregnancies at 18 weeks 0 days to 22 weeks 6 days’ gestation.

Data collection

**Figure 1:**
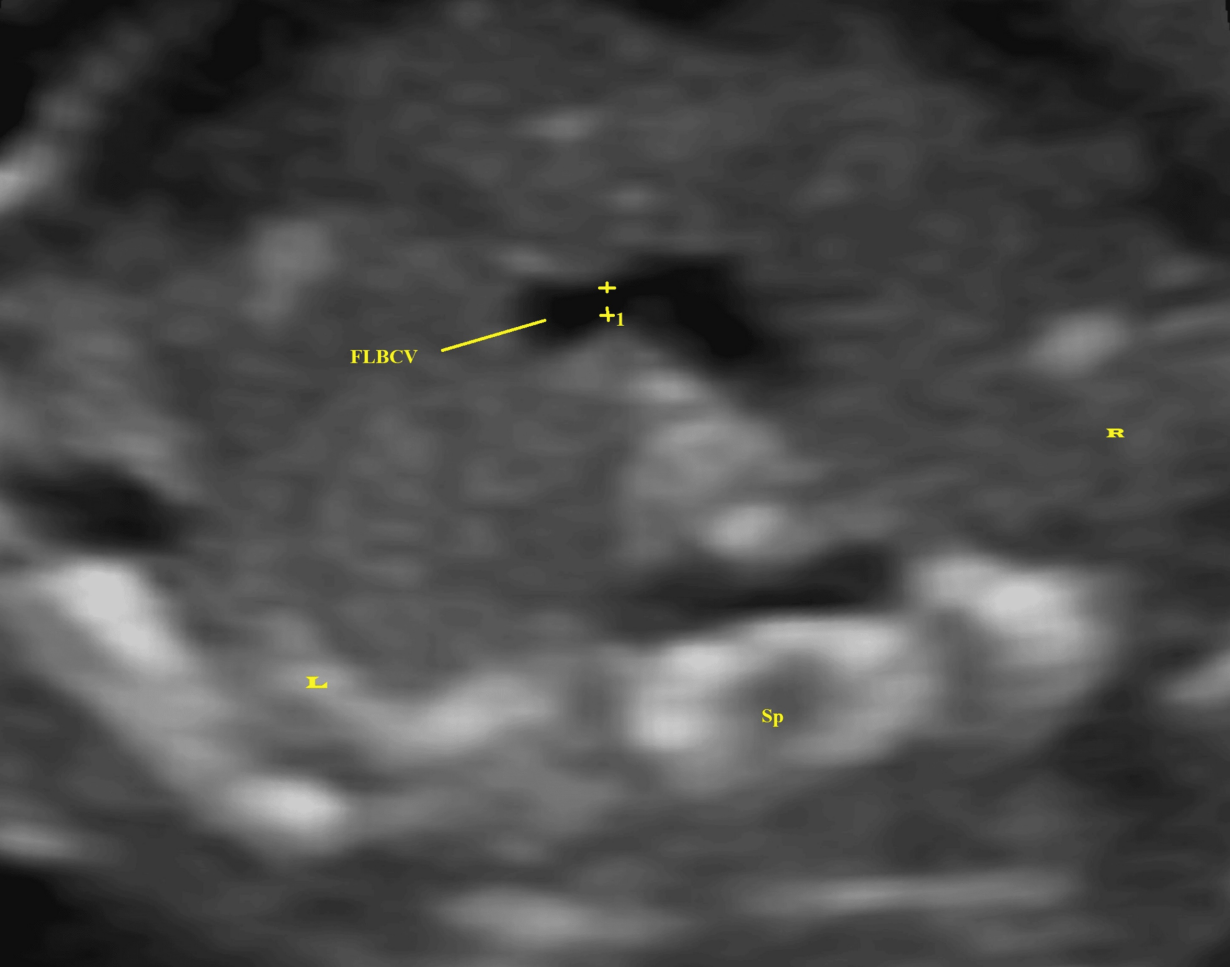
2D ultrasonographic image of the fetus showing the anteroposterior diameter of the FLBCV (1) FLBCV: fetal left brachiocephalic vein, Sp: spine, L: left, R: right, 2D: two dimensional

Data recorded for each patient included maternal height, weight, BMI, last menstrual period from the maternal case sheet, fetal biparietal diameter (BPD), head circumference (HC), abdominal circumference (AC), femur length (FL), gestational age (GA) by ultrasound, and anteroposterior diameter of the FLBCV. These were recorded by performing transabdominal ultrasound using a GE Logic P9 ultrasound machine (GE Healthcare, Wauwatosa, WI 53226, USA) with a C1-5-RS convex (1-5 MHz) probe. First, 300 eligible participants underwent basic fetal echocardiography following ISUOG guidelines (2013) [5], with documentation of cardiac planes in a digital image database. Of these, 13 patients were excluded from analysis because the fetuses had major cardiac anomalies (four with ventricular septal defect, three with atrial septal defect, and one with atrioventricular canal defect), and in five patients, the sonologist was unable to locate the LBCV in its normal position in the superior mediastinum (due to absence and three due to abnormal position and course). In 287 patients, the FLBCV was identified in a dorsoposterior fetal position by first visualizing the three vessels and trachea (3VT) view and then adjusting the transducer cranially and obliquely to the left. The anteroposterior diameter of the LBCV was measured in its middle part, anterior to the spine, using 2D imaging (as shown in Figure 1).

**Figure 2:**
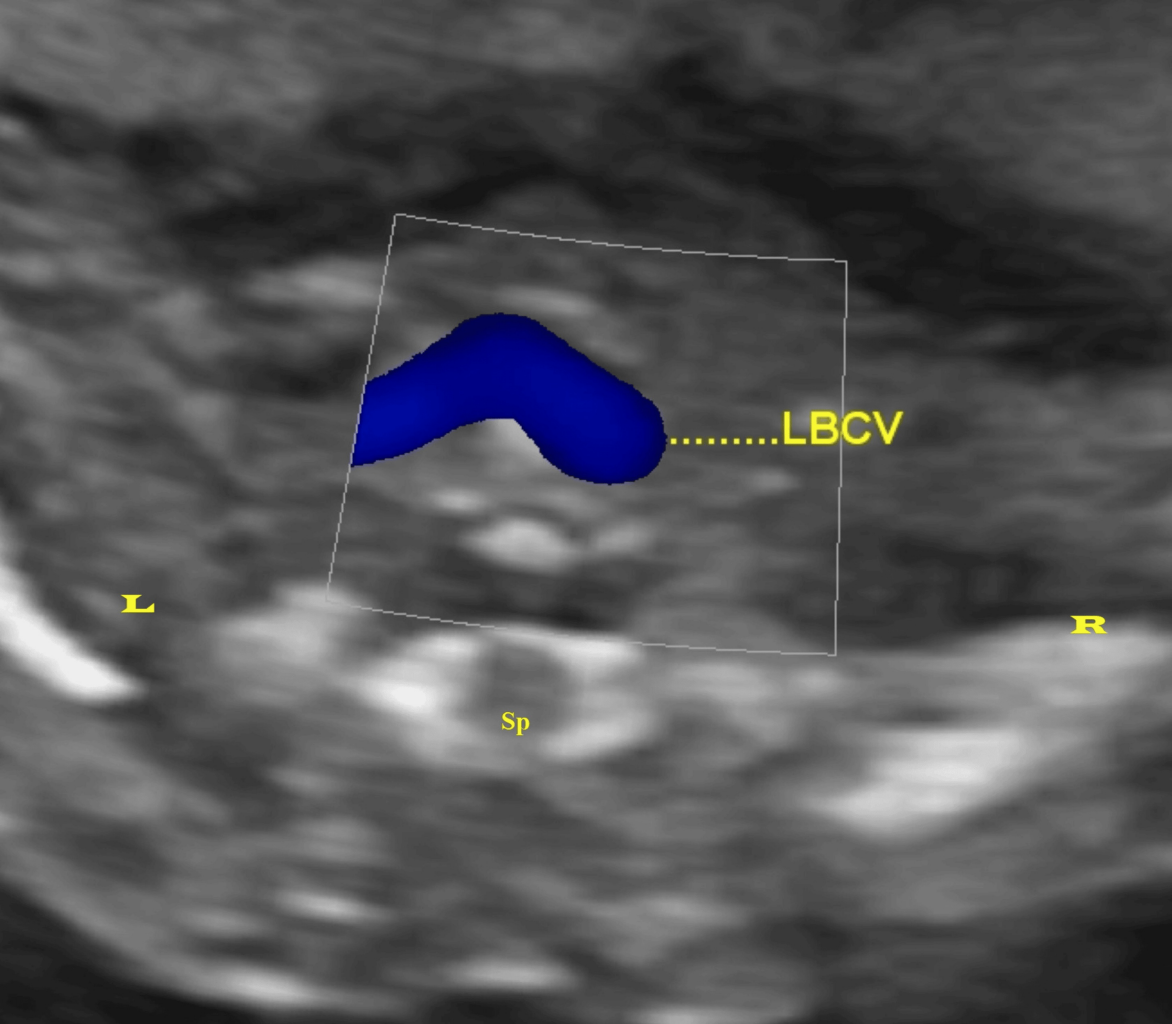
Color doppler ultrasound imaging of the FLBCV LBCV: left brachiocephalic vein, FLBCV: fetal left brachiocephalic vein, Sp: spine, L: left, R: right

Each measurement was taken three times, and the average was used for further analysis. Measurements were taken in the absence of fetal respiratory movement to avoid alterations due to changes in venous return with respiration. Color Doppler ultrasound was employed to confirm the LBCV crossing from the left to the right side of the thorax (as indicated by the blue color of the venous flow in Figure 2).

Statistical analysis

Data analysis was performed using the statistical software Jamovi 2.5.6 (retrieved from https://www.jamovi.org). A percentile chart was used to denote the normative values (ranging from 5th to 95th percentile values) of FLBCV diameter across GA 18 weeks 0 days to 22 weeks six days. The dispersion of the values was depicted by a scatter plot. We also examined the correlations between FLBCV diameter and various maternal and fetal parameters. Spearman’s correlation coefficient was used to determine the strength and direction of linear relationships. A linear regression analysis was performed to investigate the predictors of FLBCV diameter. A p-value of <0.05 was considered statistically significant.

## Results

The anteroposterior diameter of the FLBCV was measured by ultrasound in 287 fetuses between 18 weeks 0 days and 22 weeks six days of gestation. This diameter increased throughout the pregnancy, with a median value of 1.87 mm with an interquartile range (IQR) of 1.82-1.92 mm at 18 completed weeks to 2.27 mm with an IQR of 2.21-2.33 mm at 22 completed weeks of gestation. Table 1 shows the 5th, median (with IQR), and 95th percentile values of FLBCV diameter at different weeks of gestation.

**Table 1:** FLBCV diameter at different weeks of gestation FLBCV: fetal left brachiocephalic vein, mm: millimeter, IQR: interquartile range, wk: week, d: day, GA: gestational age

GA	FLBCV diameter (mm) 5th percentile	FLBCV diameter (mm) median (IQR)	FLBCV diameter (mm) 95th percentile
22 wk 0 d to 22 wk 6 d	2.02	2.27 (2.21-2.33)	2.34
21 wk 0 d to 21 wk 6 d	1.97	2.17 (2.07-2.27)	2.37
20 wk 0 d to 20 wk 6 d	1.90	2.08 (1.95-2.21)	2.33
19 wk 0 d to 19 wk 6 d	1.83	2.0 (1.90-2.10)	2.27
18 wk 0 d to 18 wk 6 d	1.77	1.87 (1.82-1.92)	2.02

**Figure 3:**
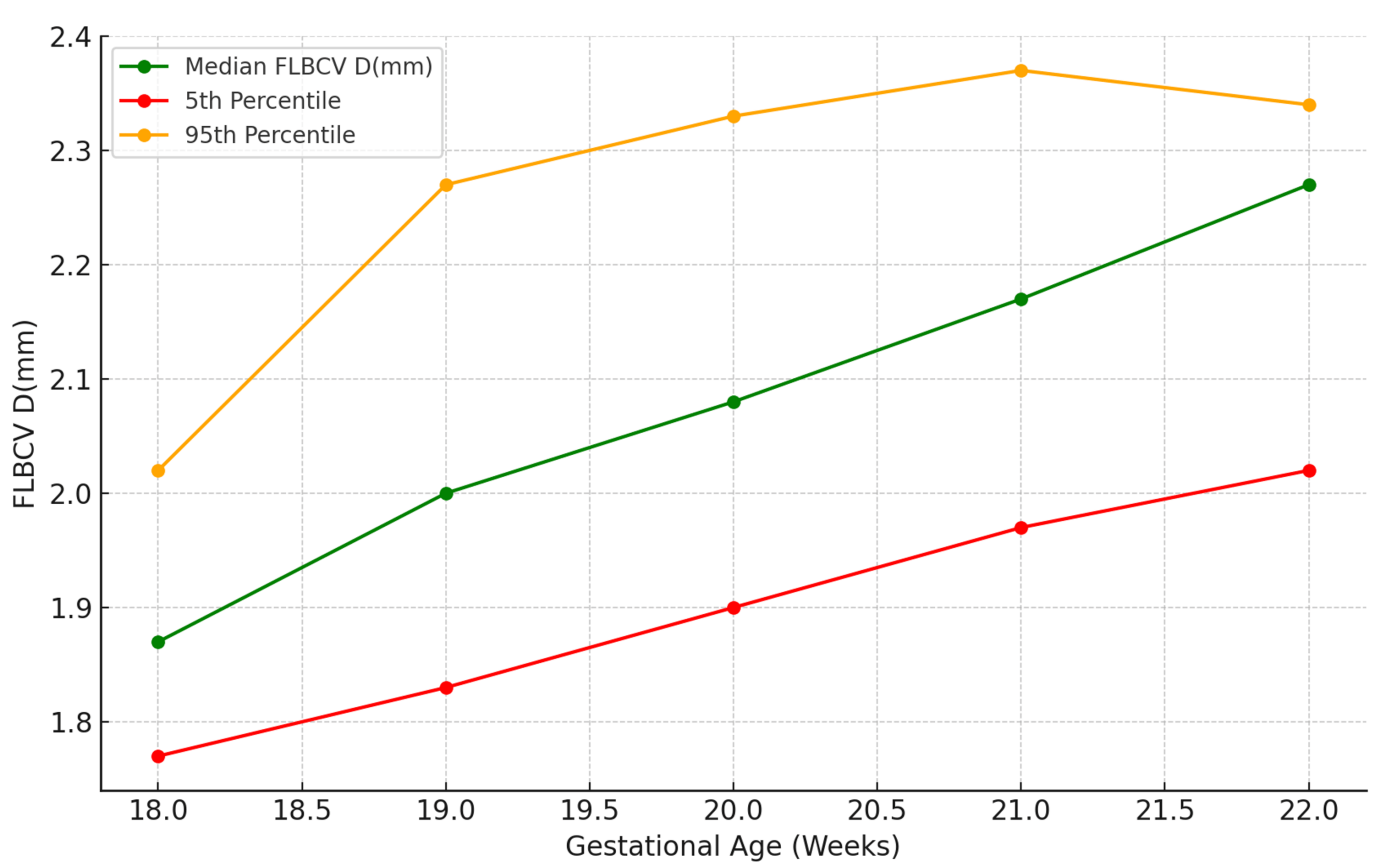
Percentile chart of FLBCV diameter across different GA FLBCV D: fetal left brachiocephalic vein diameter, mm: millimeter, GA: gestational age

Figure 3 illustrates the percentile distribution of FLBCV diameter measured in millimeters (mm) across different GA in weeks, ranging from 18 weeks 0 days to 22 weeks six days. The data is represented through three key percentiles: the fifth percentile (red), median (green), and 95th percentile (orange). The trend indicates a consistent increase in FLBCV diameter as the GA progresses, with the 95th percentile showing a more pronounced growth, particularly between 18 and 20 weeks, after which the rate of increase slightly stabilizes. The median values also exhibit a steady upward trend, while the fifth percentile grows at a more gradual pace. This chart is crucial for understanding the normal range of FLBCV development during mid-pregnancy, serving as a reference for identifying deviations in fetal circulation.

**Figure 4:**
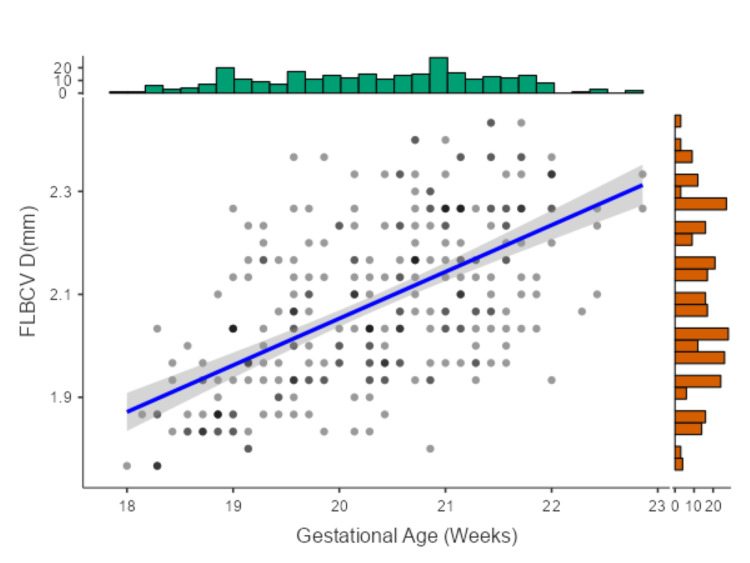
Scatter plot showing the anteroposterior diameter of the FLBCV in 287 singleton fetuses of 18-23 weeks of gestation FLBCV D: fetal left brachiocephalic vein diameter, mm: millimeter

Figure 4 depicts FLBCV diameter (mm) for a sample of fetuses aged between 18 weeks 0 days to 22 weeks six days. The plot includes a linear regression line with a shaded confidence interval, as well as histograms showing the distribution of GA and FLBCV diameter.

In our study, we analyzed the relationships between various maternal anthropometric and fetal biometric parameters related to FLBCV diameter during 18-23 weeks of gestation. The results are summarized in Table 2.

**Table 2:** Correlation analysis between various fetal biometric parameters and maternal anthropometric measurements with FLBCV diameter FLBCV: fetal left brachiocephalic vein, BPD: biparietal diameter, HC: head circumference, AC: abdominal circumference, FL: femur length, cm: centimeter, Kg: kilogram, Kg/m^2^: kilogram/meter^2^ * Spearman's rank-order correlation test was used to analyze the p-value

Variable	Spearman’s rho (correlation coefficient)	p-value*	Interpretation
BPD (cm)	0.54	<0.001	Moderate positive correlation. Larger BPD is associated with a larger FLBCV diameter.
HC (cm)	0.55	<0.001	Moderate positive correlation. Larger HC is associated with a larger FLBCV diameter.
AC (cm)	0.49	<0.001	Moderate positive correlation. Larger AC is associated with a larger FLBCV diameter.
FL (cm)	0.47	<0.001	Moderate positive correlation. Longer FL is associated with a larger FLBCV diameter.
Maternal weight (Kg)	0.12	0.046	Weak positive correlation. Heavier maternal weight is slightly associated with a larger FLBCV diameter.
Maternal height (cm)	0.05	0.384	Very weak and non-significant correlation. No significant influence of maternal height on FLBCV diameter.
Maternal BMI (Kg/m^2^)	0.14	0.022	Weak positive correlation. Higher maternal BMI is slightly associated with a larger FLBCV diameter.

A linear regression analysis was performed to explore the predictors of FLBCV diameter, which (as shown in Table 3) highlights the HC as the most significant predictor of FLBCV diameter, with a positive and statistically significant relationship. While BPD shows a positive trend, it does not reach statistical significance. AC and FL do not appear to be significant predictors of FLBCV diameter. These findings suggest that HC is a critical factor in predicting FLBCV diameter, potentially offering insights into fetal development and vascular dimensions.

**Table 3:** Linear regression analysis of the fetal biometric predictors for the LBCV diameter FLBCV D: fetal left brachiocephalic vein diameter, LBCV: left brachiocephalic vein, mm: millimeter, BPD: biparietal diameter, HC: head circumference, AC: abdominal circumference, FL: femur length, SE: standard error * Statistical test used to analyze p*-*values: t-test for regression coefficients, ** most significant

Model coefficients - FLBCV D (mm)				
Predictor	Estimate	SE	t	p-value*
Intercept	0.87	0.11	8.21	<0.001
BPD	0.09	0.05	1.76	0.079
HC	0.04	0.01	2.73	0.007**
AC	0.01	0.01	0.63	0.53
FL	-0.01	0.03	-0.28	0.781

## Discussion

LBCV abnormalities are uncommon [7,8] and poorly understood in utero. There has been a description of a correlation between extracardiac, genetic, and congenital heart defects and dilated LBCV [1,6,9]. However, sometimes dilatation of the LBCV is transient and usually resolves with time without leaving any significant clinical impact [7]. In our study, among 300 singleton pregnancies screened, in five fetuses, the LBCV was not in its actual position (two due to absence and three due to abnormal position and course), which also aligns with previous studies [9,10].

Although the intra-thymic form of the LBCV appears to be merely an isolated variant of the systemic venous return with no clinical significance, the subaortic, retroesophageal, dilated, and absent forms appeared potentially associated with cardiac and genetic abnormalities. With dilatation of the LBCV, the likelihood of pulmonary venous return anomalies or arteriovenous malformations increases [9,11]. In our study, after excluding those fetuses with absent or aberrant LBCV, we established normative values for fetal LBCV diameter in our population, which closely aligns with the results obtained by the study done by Kaymak et al. [12]. These reference values are crucial for identifying abnormal LBCV diameter, which may be indicative of underlying cardiac and extracardiac anomalies (such as TAPVC, coarctation of the aorta, arteriovenous malformation, etc.) [6].

Our study established significant correlations between FLBCV diameter and key fetal biometric measurements, particularly highlighting HC as a critical predictor. Unlike previous studies, which have not thoroughly explored the relationship between FLBCV dimensions and these fetal parameters, our findings offered a novel perspective on fetal growth assessment. The weaker correlations observed with maternal characteristics such as weight and BMI further underscore the direct influence of fetal metrics on FLBCV diameter. These insights open avenues for further research to probe into the mechanisms driving these associations and their implications for fetal cardiovascular development.

Eastern India, with its unique demographic and epidemiological characteristics, represents a significant but under-researched population in this context. Tertiary care hospitals in this region serve a large number of patients from varied socio-economic backgrounds, providing an ideal setting for generating normative data that are both representative and clinically relevant. Ultrasound measurements were performed by one experienced examiner, thereby eliminating inter-observer variations. It is one of the strengths of our study. However, there are a few limitations to our study. As a single-center study, its findings may not be generalizable. Furthermore, the relatively small sample size limits the statistical power and may affect the robustness of the conclusions. Future research with a larger, multi-center sample could provide more comprehensive data and strengthen the applicability of the findings across different settings.

## Conclusions

This study provides essential normative data on FLBCV dimensions in the Indian population, offering a valuable reference for clinicians in prenatal diagnostics. The incorporation of LBCV measurements in routine fetal echocardiography may enhance the detection of congenital anomalies, contributing to improved prenatal care.
